# An engineered PD1-Fc fusion produced in *N. benthamiana* plants efficiently blocks PD1/PDL1 interaction

**DOI:** 10.1007/s00299-025-03475-0

**Published:** 2025-03-22

**Authors:** Shiva Izadi, Rafaela Abrantes, Simon Gumpelmair, Vinny Kunnummel, Henrique O. Duarte, Peter Steinberger, Celso A. Reis, Alexandra Castilho

**Affiliations:** 1 Department of Biotechnology and Food Science Institute of Plant Biotechnology and Cell Biology, BOKU University, Muthgasse 18, 1190 Vienna, Austria; 2 i3S Instituto de Investigação e Inovação em Saúde, Universidade do Porto, Porto, Portugal; 3 Institute of Molecular Pathology and Immunology, University of Porto (IPATIMUP), Porto, Portugal; 4 Instituto de Ciências Biomédicas Abel Salazar (ICBAS), Universidade do Porto, Porto, Portugal; 5 Division of Immune Receptors and T Cell Activation, Institute of Immunology, Center for Pathophysiology, Infectiology and Immunology, Medical University of Vienna, Vienna, Austria; 6 Faculty of Medicine (FMUP), University of Porto, Porto, Portugal

**Keywords:** Checkpoint inhibitors, PD1/PDL1, *Nicotiana benthamiana*, Fc fusions, Protein glycosylation

## Abstract

**Key message:**

**Plant-made PD1–Fc fusions engineered for optimized glycosylation and Fc-receptor engagement are highly efficient in blocking PD1/PDL1 interactions and can be cost-effective alternatives to antibody-based immune checkpoint inhibitors.**

**Abstract:**

Immune checkpoint inhibitors (ICIs) are antibodies to receptors that have pivotal roles during T-cell activation processes. The programmed cell death 1 (PD1) can be regarded as the primary immune checkpoint and antibodies targeting PD1 or its ligand PDL1 have revolutionized immunotherapy of cancer. However, the majority of patients fail to respond, and treatment resistance as well as immune-related adverse events are commonly associated with this therapy. Alternatives to antibody-based ICIs targeting the PD1 pathway may bear the potential to overcome some of these shortcomings. Here, we have used a plant expression platform based on the tobacco relative *Nicotiana benthamiana* to generate immunoglobulin fusion proteins harboring the wild type or an affinity-enhanced PD1 ectodomain. We have exploited the versatility of our system to generate variants that differed regarding their glycosylation profile as well as their capability to engage Fc-receptors. Unlike its wild-type counterpart, the affinity-enhanced versions showed strongly augmented capabilities to engage PDL1 in both protein- and cell-based assays. Moreover, in contrast with clinical antibodies, their binding is not affected by the glycosylation status of PDL1. Importantly, we could demonstrate that the plant-made PD1 fusion proteins are highly efficient in blocking inhibitory PD1 signaling in a T cell reporter assay. Taken together, our study highlights the utility of our plant-based protein expression platform to generate biologics with therapeutic potential. Targeting PDL1 with plant derived affinity-enhanced PD1 immunoglobulin fusion proteins may reduce overstimulation associated with antibody-based therapies while retaining favorable features of ICIs such as long serum half-life.

**Supplementary Information:**

The online version contains supplementary material available at 10.1007/s00299-025-03475-0.

## Introduction

Tumor immune escape remains an undisputable hurdle in the development of effective anticancer therapies. It has been recognized that cancer cells can escape immune surveillance and antitumor immunity by several mechanisms, which promote tumor progression and resistance to immunotherapy (Bagchi et al. 2021; Mitra et al. 2024).

Immune checkpoints are molecules or pathways that regulate the activation and function of the immune system. These membrane proteins expressed on effector cells mediate multiple co-inhibitory and co-stimulatory pathways (Ghosh et al. 2021; Parvez et al. 2023). One of the best-known immune checkpoint pathways is the programmed cell death 1 (PD1)/programmed cell death 1 ligand (PDL1) axis. PD1 is a monomeric type I immunosuppressive checkpoint receptor expressed on the surface of various immune cells (e.g., T/B cells and natural killer (NK) cells) that acts as a “off switch” preventing T cells from attacking other cells and limiting autoimmunity (Sharpe and Pauken 2018). It does this when it engages with its ligands (PDL1 and PDL2), transmitting inhibitory signals that suppress T-cell function (Chen et al. 2020; Ghosh et al. 2021; Parvez et al. 2023). Tumor cells take advantage of these natural protective inhibitory mechanisms by upregulating the PDL1 which actively support cancer cell evasion from immune surveillance (Zou and Chen 2008; Han et al. 2020). Therefore, the PDL1/PD1 checkpoint axis has emerged as a valuable therapeutic target. Immune checkpoint inhibitors (ICIs) are a class of drugs that block the PD1/PDL1 interaction, thereby preventing inhibitory PD1 signaling and empowering T cells to effectively eliminate cancer cells (He and Xu 2020).

Six antibodies targeting the PD1/PDL1 axis have already been approved by the US Food and Drug Administration (FDA) for clinical use in several cancer settings and numerous others are currently under clinical development (Gong et al. 2018; Jiang et al. 2020; Vaddepally et al. 2020; Waldman et al. 2020; Twomey and Zhang 2021; Parvez et al. 2023).

Despite the great success of ICIs targeting PD1/PDL1 in the management of a broad range of cancer types, there are inherent drawbacks and limitations such as poor penetration in tissues and tumors, and the occurrence of immune-related adverse events (AEs). Antibody-induced AEs can be due to excessive immune activation and off-target binding leading to unexpected immune responses.

Antibodies to PD1 and PDL1 have different requirements for FcγR engagement to attain optimal antitumor activity (Yu et al. 2020; Dahan and Korman 2024). In particular, the role of the Fc-mediated effector functions in cancer therapy using PDL1 blocking antibodies has been discussed controversially (Leitner et al. 2021; Yu et al. 2020; Cohen Saban et al. 2023). With the exception of avelumab, PDL1 blockers are IgG1 monoclonal antibodies (mAbs) with reduced Fc-effector function via amino acid substitutions on the FcγR-binding domain (durvalumab) or “null” glycosylation (atezolizumab). In contrast, avelumab (PDL1 mAb) retains the native IgG1-Fc region, which is capable of engaging Fcγ receptors on NK cells to induce antibody-dependent cellular cytotoxicity (ADCC) as a part of its mechanism of action, allowing it to elicit an immune response against PDL1^+^ tumors (Hamilton and Rath 2017).

It is well known that the conserved *N-*glycan in the Fc region of mAbs impacts their interaction with FcγRs and the resulting effector functions (Archer et al. 2022). Recently, an engineered afucosylated avelumab optimized for enhanced FcγRIIIA engagement was shown to induce stronger antitumor immune responses compared with the parental antibody (Cohen Saban et al. 2023).

While antibody-based PD1/PDL1 inhibitors have expanded the patient’s treatment options, the response rates are limited, and some initial responders resist treatment over time. The mechanisms leading to both primary and acquired resistance to PD1/PDL1 inhibition are varied and multifactorial (Nowicki et al. 2018; Lei et al. 2020; Pathak et al. 2020; Mitra et al. 2024). The clinical performance of therapeutic antibodies can be severely hampered by molecular resistance. Over the past years, it has become evident that altered glycosylation, a hallmark in cancer cells, plays a key role in the acquisition of resistance to therapeutic agents (Mereiter et al. 2019). In particular, hypersialylation of cancer receptors is a common cancer glyco-signature that hinders the affinity of targeted antibodies (Rodrigues and Macauley 2018; Munkley 2022). Indeed, recent findings suggest that the structural hindrance cause by the heavy glycosylation of PDL1 hampers antigen recognition by PDL1 antibodies, which was significantly improved after enzymatic deglycosylation with peptide-N-glycosidase F (PNGase F) (Lee et al. 2019; Dressler et al. 2022) or removal of terminal sialic acid residues by sialidases (Zhou et al. 2021). As the mechanisms of resistance to PD1/PDL1 blockade continue to be further characterized, new strategies are being developed that can potentially improve treatment outcomes.

An alternative approach to antagonize the PD1/PDL1 axis is to use a soluble PD1 ectodomain acting as a ligand trap or decoy to neutralize PDL1-expressing cancer cells and therefore hijack the process of immune suppression by tumor cells (Stanczak et al. 2018).

The low affinity between native PD1 and PDL1 has hindered the design of powerful PD1-based inhibitors by mimicry (Yin et al. 2021). However, recent studies have reported on engineered PD1 variants with increased affinity to PDL1 showing its great potential in cancer immunotherapy (Maute et al. 2015; Li et al. 2018b). Of note, NULOJIX® (*belatacept*)*,* an Fc fusion protein based on CTLA-4, is already in use, demonstrating the potential of biologics based on affinity-enhanced immune checkpoints in the clinics (Vincenti 2016).

Plant-based expression platforms have emerged as cost-effective alternatives for the production high-quality proteins for research, diagnostic, and therapeutic applications. One of the key advantages of plant-based expression platforms lies in their scalability and cost-effectiveness, allowing the production of complex proteins at a fraction of the cost compared to conventional methods. Additionally, with the optimization of the transient expression systems, tailored recombinant proteins can be produced in a manner of weeks (Zahmanova et al. 2023; Jadhav and Khare 2024). The tobacco relative, *N. benthamiana,* is the most frequently used plant in molecular farming (PMF) to produce recombinant proteins using transient expression. Recently, we used this system to produce variants of durvalumab with therapeutically favorable FcR engagement. Plant-made durvalumab formulations were able to bind to PDL1^+^-cancer cells and efficiently block the PD1/PDL1 interaction (Izadi et al. 2024).

Here, we set up to use PMF to produce wild-type (^WT^PD1) and affinity-optimized versions of the extracellular domain of PD1 (soluble PD1) and compared their ability to bind to PD1 ligand (PDL1) expressed in gastrointestinal cancer cells and to act as inhibitors (competitors) of the natural PD1/PDL1 interaction. In addition, we generated glyco-variants of the PDL1 to assess the impact of PDL1 glycosylation on its recognition by soluble PD1. ^WT^PD1 and PD1 were expressed as fusions to the crystallizable region (Fc) of human IgGs. Fc fusion proteins have been successfully developed for therapeutic purposes (Jafari et al. 2017). Apart from increasing protein stability and avidity due to dimerization, the Fc domain confers functional properties of the parental antibody, such as engaging with the FcγR pathways. We generated PD1-Fc formulations (i) with and without Fc-mediated effector functions (Izadi et al. 2024); (ii) with different glycosylation profiles, and (iii) in a dimeric and monomeric format. Our study identifies plant-made PD1-Fc variants with a strong capability to disrupt PD1–PDL1 interaction and to block PD1 signaling which might have potential as alternative to therapeutic antibodies targeting the PD1 axis.

## Material and methods

### Construction of the PD1-Fc protein fusions

**Fig. 1:**
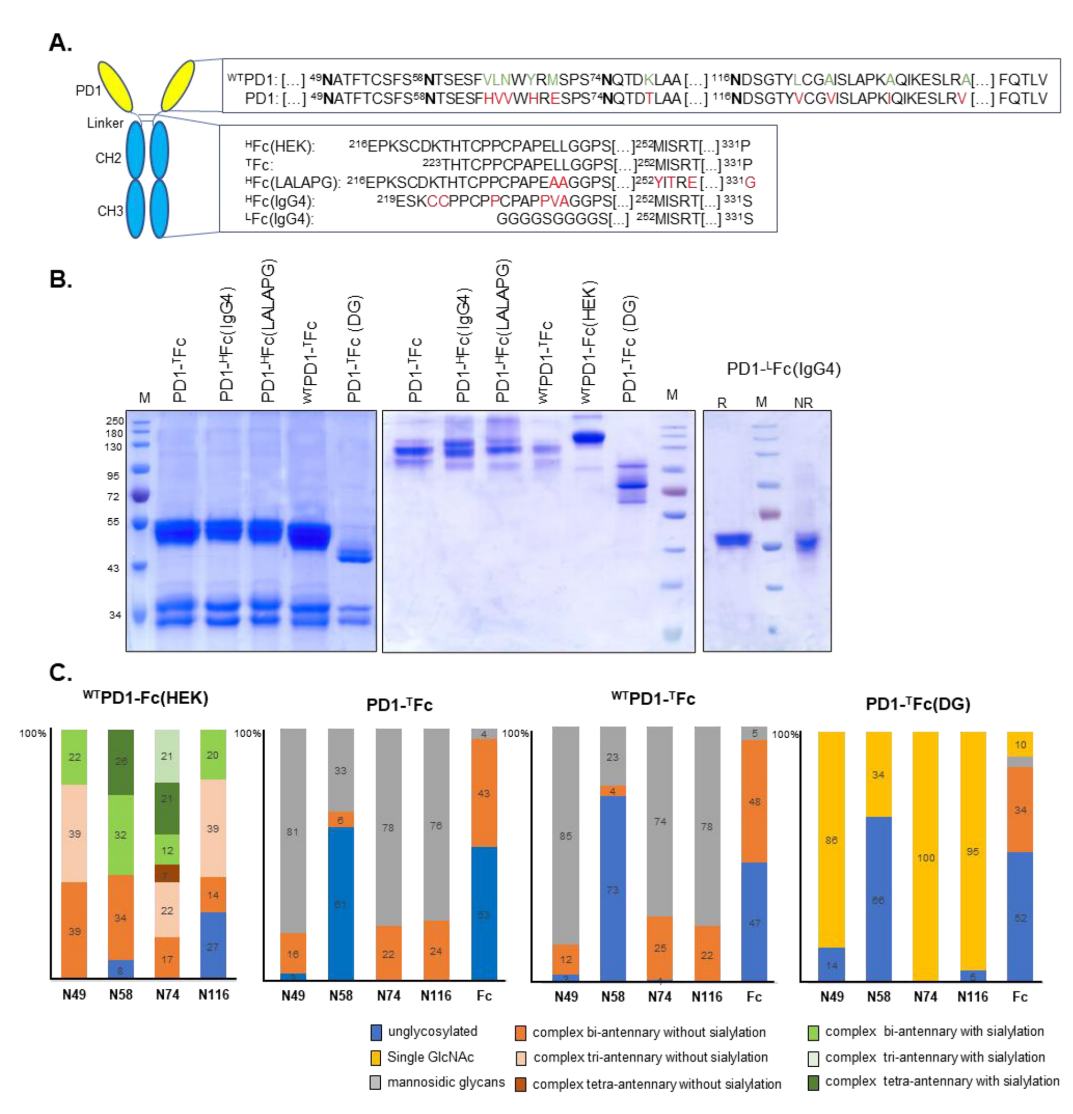
Expression and glycoengineering of PD1-Fc variants. **A** Schematic representation of a PD1-Fc fusion highlighting the differences in amino acid sequence between ^WT^PD1 and PD1 and within the hinge and Fc domains. PD1 glycosylation sites are marked in bold. **B** PD1-Fc variants expressed in *N. benthamiana* ΔXF plants and purified by affinity chromatography with protein A were analyzed by SDS-PAGE gels stained with CBB in reducing conditions (left panel) or in non-reducing conditions after size-exclusion chromatography (middle panel). Reduced and non-reduced CBB staining of purified monomeric PD1-^L^Fc(IgG4) fusion (right panel). The apparent molecular mass of marker proteins (M) is shown in kilo Dalton (kDa). **C** Relative abundance of glycoforms detected on PD1-Fc variants. Percentages are mean values of experiments carried out in three repetitions. For detailed information see Fig. S1

The extracellular domain of the human PD1 (Q15116, aa 25–170) was codon optimized for *N. benthamiana.* The PD1 was synthetized with ten amino acid substitutions known to increase affinity to PDL1 (Maute et al. 2015). In addition, we synthetized a wild-type sequence of PD1 (^WT^PD1) (Fig. 1). PD1 and ^WT^PD1 were fused to the Fc domain of IgG antibodies to generate different PD1-Fc formulations. We previously reported the construction of IgG heavy chain variants to generate durvalumab (IMFINZI^®^) as a wild-type IgG1 and its ‘Fc-effector-silent’ variant (LALAPG: L234A/L235A/P331G) carrying further modifications to increase antibody half-life (YTE: M252Y/S254T/T256E) and an IgG4 with six substitutions (S228P/Y219C/G220C/ E233P/F234V/P235A) for protein stabilization and reduced affinity to FcγRI (Izadi et al. 2024).

Fc fragments amplified from these constructs were cloned into the magnICON® tobacco mosaic virus-based vectors (TMVα: pICHα26211, Klimyuk et al. 2014) that include a signal peptide to target proteins to the secretory pathway and two *Bsa*I restriction sites. This resulted in the generation of Fc fusion cloning vectors carrying a wild-type IgG1-Fc with a truncated hinge region (^T^Fc); an IgG1 “Fc-effector-silent’ with a full hinge region [^H^Fc(LALAPG)] and an IgG4-Fc with a mutated hinge region [^H^Fc(IgG4)]. In addition, the hinge region of ^H^Fc(IgG4) was substituted for a flexible linker (Gly-Gly-Gly-Gly-Ser)_2_ to create the ^L^Fc(IgG4) fusion cloning vector (Fig. 1). Two upstream *Bsa*I sites were used to clone in PD1 DNA fragments generating the expression vectors: PD1-^T^Fc, PD1-^H^Fc(LALAPG), PD1-^H^Fc(IgG4), and PD1-^L^Fc(IgG4). In addition, the ^WT^PD1 was cloned into ^T^Fc, generating the ^WT^PD1-^T^Fc expression vector.

A PD1-Fc fusion expressed in HEK293 cells [^WT^PD1-Fc(HEK)] was purchased from R&D (1086-PD-050).

### Cloning of PDL1

Cloning of the human programmed cell death 1 ligand (PDL1) with a C-terminal polyhistidine tag (PDL1_His_) was described elsewhere (Izadi et al. 2024). Additionally, the extracellular domain (aa 18–283, Q9NZQ7) of PDL1 was fused to the constant domain of the human kappa light chain (CAR58102) lacking the terminal cysteine (Gattinger et al. 2021) and cloned into the magnICON^®^ potato virus-X vector (PVXα: pICHα31150, Klimyuk et al. 2014) (PDL1-mLc).

### Transient expression and glycoengineering of PD1 and PDL1

Recombinant proteins were transiently expressed in *N. benthamiana* glycosylation mutant plants, ΔXF (Strasser et al. 2008) via agroinfiltration. Bacterial cultures used to express PD1-Fc and PDL1 were infiltrated at an optical density (OD_600_) of 0.3, while those to deliver genes for glycoengineering were infiltrated at OD_600_ of 0.1. In co-expression experiments, TMVαPD1-Fc and PVXαPDL1-mLc bacterial cultures were mixed 1:1.

Infiltrated leaves harvested 4 days post-infiltration (dpi) were used for total soluble protein extraction (PD1-Fc and PDL1-mLc) or apoplastic fluid collection (PDL1_His_).

PDL1 was produced as two glyco-variants: expression of PDL1 in *N. benthamiana* ΔXF leads to the generation of afucosylated proteins, while terminal α2,6-sialylation was achieved by the co-expression of genes necessary for (i) biosynthesis, (ii) activation, (iii) transport, and (iv) transfer of Neu5Ac to terminal galactose (Castilho et al. 2010; Izadi et al. 2023a). Finally, PD1 carrying a single GlcNAc residue was generated by *in planta* deglycosylation (DG) through co-expression of an endoglycosidase (EndoH) as described previously [PD1-^T^Fc(DG)] (Izadi et al. 2023b). As PDL1 expressed in ΔXF plants carry complex glycans, deglycosylation was achieved by co-expression of EndoH and a α-mannosidase inhibitor, kifunensine (20 µM), to induce the accumulation of mannosidic *N*-glycans (Izadi et al. 2023b).

### Apoplastic fluid collection

To collect proteins secreted into the apoplastic fluid (AF), infiltrated leaves were immersed in buffer solution (20 mM Na_2_HPO_4_, 100 mM NaCl, pH 7.4) and subjected to vacuum (2 × 5 min). Leaves were centrifuged (1000 rpm for 15 min) in 50 mL tubes, containing a supported mesh to allow separation of the AF from the leaf material (Castilho and Steinkellner 2016). On average, 1 g of leaf material renders approximately 650 µL of AF.

### Total soluble protein extraction

Infiltrated plant leaf material was ground using a kitchen mixer with 4 mL/g of extraction buffer (50 mM Tris/HCl, 150 mM NaCl, supplemented with 40 mM ascorbic acid and 4% PVPP, pH 7.4) at 4 ℃. The slurry was incubated for 30 min at 4 °C, centrifuged (11 000 rpm for 20 min at 4 ℃), and filtered through two layers of paper filters. The filtrate was further clarified by a pH shift and centrifuged twice at 11 000 rpm for 20 min and at 18 000 rpm for 30 min. Finally, the extract was filtered through a 0.22 µm filter (Izadi et al. 2024).

### Protein purification

PD1-Fc variants and PDL1_His_ were purified with HiTrap Protein A HP (Cytiva) and Ni–NTA His•Bind^®^ resin (Sigma), respectively, as described previously (Izadi et al. 2024).

Purification of PDL1-mLc was performed with KappaSelect™ (GE Healthcare) according to manufacturer’s instructions (Gattinger et al. 2021).

Pooled eluates were dialyzed overnight against PBS, pH 7.4, using SnakeSkin dialysis tubing (Thermo Fisher Scientific) with a 10-kDa molecular mass cutoff. Size-exclusion chromatography (SEC) was performed on PD1-Fc samples to remove the free Fc fraction and recover full-size fusion proteins (Izadi et al. 2024).

Protein concentration was determined spectrophotometrically at a wavelength of 280 nm (NanoDrop™ 2000, Thermo Scientific) using the extinction coefficients 1.11, 1.0, and 1.02 M^−1^ cm^−1^ for PD1-Fc variants, PDL1_His_, and PDL1-mLc, respectively.

### Co-immunoprecipitation

 ~ 3 µg of dimeric of PD1-Fc variants were used to spike 1 mL of apoplastic fluid collected from leaves infiltrated with PDL1_His_. Proteins were purified with Ni–NTA His•Bind beads and analyzed by immunoblotting.

### Protein analysis

Purified recombinant proteins were fractionated by 10% SDS-PAGE under reducing and non-reducing conditions and either stained with Coomassie brilliant blue (CBB, G-250) or analyzed by immunoblotting.

PD1-Fc, PDL1-mLc, and PDL1_His_ were analyzed by Western blot using anti-human gamma chain- HRP (1:5000, Sigma-Aldrich), anti-human kappa-chain-HRP (1:5000, Sigma-Aldrich), and mouse anti-6xHis (1:10,000, Clontech Mountain View) antibodies, respectively. PDL1_His_ was also analyzed by lectin blotting with biotinylated *Sambucus nigra* agglutinin (SNA, Vector, 5 μg/mL in PBST) and HRP-conjugated streptavidin (1:10,000, Vector).

Detection was performed with Clarity™ Western enhanced chemiluminescence reagents (Bio-Rad) and images were captured with a Fusion Solo S image system (Vilber Lourmat).

### Mass spectrometry-based glycomic analysis

PD1-Fc fusions and PDL1 were digested in solution with chymotrypsin and trypsin, respectively, and subjected to LC–ESI–MS analysis (Grunwald-Gruber et al. 2017; Izadi et al. 2024).

### Enzyme-linked immunosorbent assays (ELISA)

The binding affinity of PD1-Fc variants to PDL1_His_ was determined by ELISA in three technical replicates as in Izadi et al. (2024). Briefly, serial dilutions of PD1-Fc variants (1000–0.48 ng/mL) were added to plates coated with 200 ng PDL1_His_ and detected with anti-human IgG-HRP (1:20,000, Promega). For plant-made ^WT^PD1-^T^Fc and ^WT^PD1-Fc expressed in HEK293 cells (^WT^PD1-Fc(HEK), R&D, 1086-PD-050), the plates were coated with 1 µg PDL1_His_ and the serial dilutions were from 20,000 to 10 ng/mL.

The binding of PD1-^T^Fc to PDL1_His_ glyco-variants was also assessed by ELISA. A goat anti-human gamma chain (100 ng, Sigma-Aldrich) coated on plates was used to immobilize 100 ng PD1-^T^Fc. Plates were probed with a serial dilution of PDL1_His_ glyco-variants glyco-variants(1000–0.48 ng/mL) and detected with mouse anti-6xHis (1:5000, Clontech Mountain View), followed by anti-mouse IgG-HRP (1:20,000, Promega).

Unrelated Fc fusion and His-tagged proteins were used as negative controls. Absorbance was measured at 450 nm with reference to 620 nm using a Tecan Spark^®^ spectrophotometer. Effective half-maximum concentrations (EC_50_) were defined at the infliction point of the sigmoidal curve by non-linear regression of the blank-corrected absorbance values using GraphPad Prism (version 8).

### Surface plasmon resonance (SPR)

Binding of PDL1_His_ to PD1-Fc variants was determined by surface plasmon resonance (SPR) in three replicates, using the Biacore T200 system (GE Healthcare) at 25 ℃. Immobilized human IgG1(1.25 ng/µL) on a Biacore CM5 Sensor Chip (Human antibody capture kit, Cytiva) was used to capture 1.5 µg/µL PD1-^T^Fc and ^WT^PD1-^H^Fc(HEK) to approximately 80 response units (RU). Avelumab (anti-PDL1) was used as a control. Measurements were done with serial dilutions of PDL1_His_ (analyte) in running buffer (10 mM HEPES pH7.4; 150 mM NaCl; 0.005% Tween 20). Analyte serial dilutions of 250 nM-4000 nM, 0.1 nM-1000 nM, and 0.1 nM-75 nM were used to probe the affinity of PDL1_His_ to ^WT^PD1-^H^Fc(HEK), PD1-^T^Fc and avelumab, respectively.

The chip was regenerated in 100 mM glycine (pH 9.5). Binding kinetics, *k*_*a*_ (1/Ms), *k*_*d*_ (1/s), and K_D_ (M) were calculated from global fittings using a 1:1 binding model (BIAevaluation software).

### Binding of PD1-Fc formulations to PDL1 expressed in gastrointestinal cancer cells

For cell surface labelling of carcinoma cells with PD1-Fc formulations, NCI-N87 gastric and SW48 colorectal cancer cells were seeded in six-well culture plates (5 × 10^5^ cells/well) in complete growth medium supplemented with IFNγ (40 ng/mL) for 48 h to induce the cell surface expression of PDL1. Cells were harvested and their reactivity to plant-made PD1-Fc fusions (1 µg/mL) was evaluated by flow cytometry. A detailed cell line description and flow cytometry assay can be found elsewhere (Izadi et al. 2024).

### Cell reporter assays

Culture of reporter cell lines and flow cytometry assays were as described previously (De Sousa-Linhares et al. 2019; Izadi et al. 2024).

JE6-1-NF-kB::eGFP-PD1 reporter cells (5 × 10^4^) were co-cultured with K562S-PDL1 in 96-well plates in the presence or absence of PD1-Fc fusion proteins used at 10, 1, 0.1, 0.01 µg/mL (ten-fold dilution steps). Following 24 h of co-culture, plates were measured by flow cytometry. The geometric mean of the fluorescence intensity (gMFI) of viable reporter cells was used for further analysis. K562S cells were excluded based on their expression of red fluorescent protein (RFP). Data was normalized to the eGFP expression of PD1 reporter cells stimulated under conditions where PDL1 was fully blocked (3 μg/mL of PDL1 antibody, avelumab).

### Statistics

EC_50_ values were estimated by non-linear regression based on a four-parameter logistic curve (4PL) model with GraphPad Prism (version 9). Statistical analysis was performed with multiple comparisons one- and two-way ANOVA.

## Results

### Expression and glycoengineering of PD1-Fc variants

Despite the natural affinity between the wild-type human PD1 receptor (^WT^PD1) and its ligand PDL1 being relatively weak (Cheng et al. 2013), Ring and co-workers have used directed evolution by yeast-surface display to engineer an affinity optimized PD1 variant (HAC-V) due to the formation of new hydrogen bonds and salt bridges with PDL1 (Maute et al. 2015; Pascolutti et al. 2016). This high-affinity consensus PD1 carries ten amino acid substitutions, including a mutation N49G of the glycosylation site Asn49.

Here, codon-optimized fragments comprising the extracellular domain of the ^WT^PD1 and of a mutated HAC-V (hereby PD1) carrying nine of the ten amino acid substitutions (excluding N49G mutation) were fused to the Fc region of human IgGs (Fig. 1A). ^WT^PD1 and PD1 were fused to a fully functional Fc-IgG1 separated by a truncated hinge region (^WT^PD1-^T^Fc and PD1-^T^Fc). In addition, we generated PD1-Fc variants where PD1 was fused to mutated Fcs, lacking Fcγ receptor engagement [PD1-^H^Fc(LALAPG) and PD1-^H^Fc(IgG4)]. Additionally, PD1 was fused to the Fc-IgG4 via a linker of glycine and serine residues (G_4_S)_2_ [PD1-^L^Fc(IgG4)] (Fig. 1A). PD1-Fc formulations were transiently expressed in *N. benthamiana* glycosylation mutant plants ∆XF (Strasser et al. 2008) via agroinfiltration. Recombinant proteins were purified out of total soluble proteins extracts at 4 days post-infiltration via affinity chromatography with protein A. Coomassie brilliant blue (CBB) staining of SDS-PAGE gels run under reducing conditions shows two bands representing the full-length PD1-Fc fusions (~ 52-kDa) and free Fc (~ 34-kDa). Free Fc results from the proteolytic cleavage of the fusion proteins, a common feature of Fc fusions expressed in plants (Gattinger et al. 2021) (Fig. 1B, left panel). The size discrepancy between the observed (52-kDa) and calculated mass (~ 42-kDa) for PD1-Fc fusions is due to protein *N*-glycosylation.

After isolation of the full-length dimeric fraction by size-exclusion chromatography (SEC), CBB staining of SDS-PAGE gels run under non-reducing conditions shows that all PD1-Fc fusions assemble as homodimers comparable to the commercially available ^WT^PD1-Fc produced in HEK cells [^WT^PD1-Fc(HEK)] (Fig. 1B, middle panel). Interestingly, when the hinge region separating the PD1 and Fc-IgG4 domains is substituted by a (G_4_S)_2_ linker, the PD1-^L^Fc(IgG4) fusion is stable and no free Fc is observed. Moreover, CBB staining of SDS-PAGE gels run under non-reducing conditions shows that the fusion protein is a monomer (Fig. 1B, right panel). Dimerization of IgG1 antibodies occurs via disulphide bonds in the hinge region and non-covalent interactions between amino acids at the CH3 domains (Vidarsson et al. 2014). Recently, we showed that mutations at the CH3 domain of Fc-IgG1 resulted in stable monomeric fusion proteins (Gattinger et al. 2021). In IgG4, the R409 (rather than the equivalent lysine in IgG1) results in weaker CH3–CH3 interactions (Vidarsson et al. 2014; Rispens et al. 2014), and thus protein dimerization is abrogated in PD1-^L^Fc(IgG4).

Purification yields were similar for all PD1-Fc fusions in the range of 0.24 mg/g, where up to 50% corresponds to full-length dimeric proteins.

PD1 is a highly *N*-glycosylated protein. PD1-Fcs carry four *N*-glycosylation sites on the PD1 domain (N49, N58, N74, and N116) and one at the Fc region (N297). Site-specific glycomic profiling of ^WT^PD1-Fc(HEK) shows that all glyco-sites are decorated with complex glycans, with unglycosylated peptides being detected only at N58 and N116. Glycosylation is highly heterogeneous and comprises bi-, tri-, and tetra-antennary core-fucosylated complex glycans with or without terminal sialylation (Fig. 1C and Fig. S1). In contrast, in plant-made PD1-Fc fusions, PD1 carries mostly oligomannosidic glycans (Man5-Man9), while the Fc domain is decorated with complex bi-antennary glycans without core-fucosylation and sialylation (GnGn) (Fig. 1C and Fig. S1). Oligomannosidic glycans are typical of proteins accumulating in the ER. However, the complex glycans observed on the Fc domain indicate that the fusion proteins were processed by Golgi resident enzymes via the secretory pathway and, therefore, not retained in the ER. Mannosidic glycans observed on the PD1 domain are independent from PD1 mutations (PD1 vs. ^WT^PD1) and from the Fc used in the fusions [^T^Fc, ^H^Fc(LALAPG), ^H^Fc(IgG4), and ^L^Fc(IgG4)]. All PD1 glycosites, with the exception of N58, are efficiently glycosylated. Also, more than 50% of the Fc domain is unglycosylated. Indeed, the double band observed for free Fc (~ 34-kDa) corresponds to glycosylated and unglycosylated protein (Fig. 1B, left panel).

It has been reported that PD1 glycosylation has no influence on PD1 conformation, but glycans on N58 seem to mediate the binding affinity of some therapeutic antibodies (e.g., cemiplimab and camrelizumab) (Lu et al. 2022; Liu et al. 2020; Wang et al. 2019; Chu et al. 2024). The impact of PD1 glycosylation on its interaction to PDL1 is still elusive. One study revealed that removal of PD1 glycans, specifically at N58, reduced the binding to PDL1 (Sun et al. 2020). Here, we transiently co-express PD1-^T^Fc with a bacterial endoglycosidase (EndoH) to produce PD1-^T^Fc decorated with *N*-glycans consisting of single *N*-acetylglucosamine (GlcNAc) residue [deglycosylation: PD1-^T^Fc(DG)]. EndoH was targeted to the Golgi apparatus to avoid any interference of glycan removal with concomitant protein folding (Izadi et al. 2023b). CBB staining of purified in vivo deglycosylated PD1-^T^Fc(DG) shows a clear shift in protein size (Fig. 1B, left panel). As EndoH cleaves within the chitobiose core of high mannosidic glycans, no size shift is observed on the free Fc decorated with complex glycans (Fig. 1B, left panel). Glyco-profiling of PD1-^T^Fc(DG) shows that in all PD1 sites, the mannosidic glycans were trimmed to single GlcNAc residues and glycosylation at the Fc site remains mostly unchanged (Fig. 1C and Fig. S1).

### Expression and glycoengineering of PDL1

**Fig. 2:**
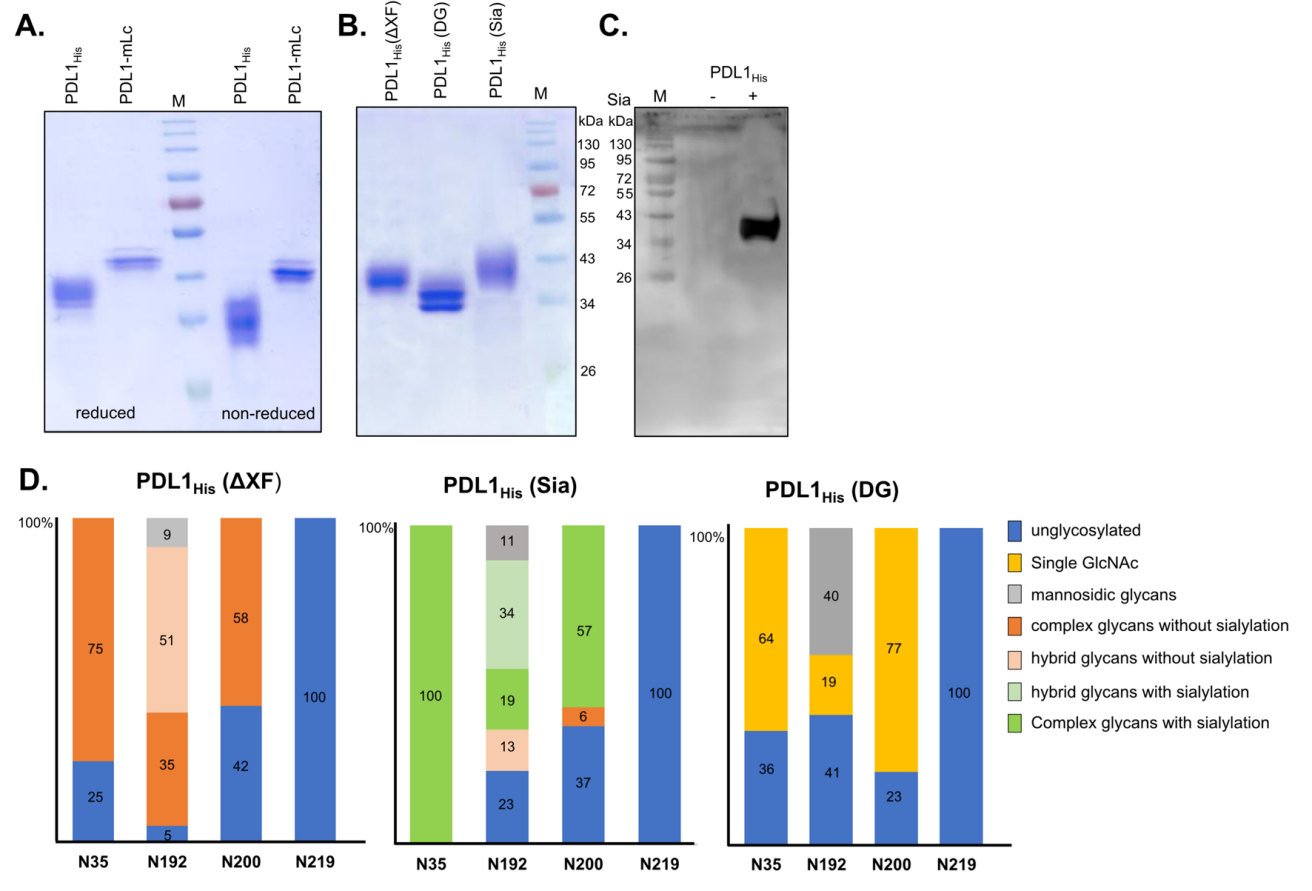
Expression and glycoengineering of PDL1. **A** PDL1_His_ and PDL1-mLc expressed in *N. benthamiana* ΔXF plants analyzed by SDS-PAGE stained with CBB under reducing and non-reducing conditions. **B** Reducing SDS-PAGE stained with CBB of purified PDL1_His_ expressed in *N. benthamiana*. PDL1_His_(ΔXF): PDL1_His_ expressed in ΔXF plants. PDL1_His_(DG): PDL1_His_ co-expressed in ΔXF plants with EndoH in the presence of kifunensine and PDL1_His_(Sia): PDL1_His_ co-expressed in ΔXF plants together with the necessary genes for protein sialylation. **C** SDS-PAGE of PDL1_His_(ΔXF) and PDL1_His_(Sia) analyzed with biotinylated *Sambucus Nigra* agglutinin (SNA) to detect the presence of terminal α2,6-sialylation of *N*-glycans. The apparent molecular mass of marker proteins (M) is shown in kilo Dalton (kDa). **D** Relative abundance of glycoforms detected on PDL1_His_ glyco-variants. Percentages are mean values of experiments carried out in two repetitions. For detailed information see Fig. S2

To evaluate the biding affinity of PD1-Fc fusions, we set up to produce recombinant PDL1. The human PDL1 consists of an ectodomain (ECD) encompassing an immunoglobulin V-like domain (IgV) and an immunoglobulin C-like domain (IgC), a transmembrane domain (TM), and a short intracellular tail (Dong et al. 2002). Previously, we cloned and expressed the ECD of PDL1 with a C*-*terminal His tag (PDL1_His_). Here, the PDL1-ECD was fused to the constant domain of a human kappa light chain (Gattinger et al. 2021). To avoid protein dimerization, we removed the terminal cysteine, generating a PDL1-mLc expression vector. Both PDL1_His_ and PDL1-mLc were transiently expressed in *N. benthamiana* ∆XF plants via agroinfiltration. Secreted proteins accumulating in the apoplastic fluid (AF) were purified with Ni–NTA His•Bind^®^ resin (PDL1_His_) or KappaSelect™ resin (PDL1-mLc) and analyzed by SDS-PAGE under reducing and non-reducing conditions (Fig. 2A). CBB staining shows that both PDL1_His_ (~ 40-kDa) and PDL1-mLc (~ 45-kDa) express as monomeric proteins. The lower size of protein bands observed in CBB staining under non-reducing conditions is due to native folded conformation of the recombinant proteins. Moreover, in reducing conditions, the apparent molecular weight of both proteins is higher than its calculated molecular mass due to extensive protein *N*-glycosylation (Fig. 2A). Indeed, PDL1 carries four potential *N*-glycosylation sites (N35, N192, N200 and N219) within the ECD.

One of the most common glycan alterations in malignant cells is hypersialylation (Munkley 2022). Glyco-profiling of PDL1 expressed in MDA-MB-231 breast cancer cells showed that PDL1 carries primarily complex sialylated glycans, with the exception of N192 that is exclusively occupied by high-mannose glycans (Benicky et al. 2021).

To evaluate the impact of PDL1 glycosylation in the binding affinity of PD1-Fc fusions, we generated PDL1_His_ glyco-variants. PDL1_His_ was produced in *N. benthamiana* ∆XF without and with co-expression of genes for (i) biosynthesis, (ii) activation, (iii) transport, and (iv) transfer of *N*-acetylneuraminic acid (Neu5Ac or Na) to terminal galactose for the synthesis of α2,6-sialylated glycans [PDL1_His_(Sia)] (Izadi et al. 2023a). In addition, *in planta* deglycosylation of PDL1_His_ was performed by co-expressing EndoH and the α-mannosidase inhibitor kifunensine (20 µM) to induce the accumulation of EndoH-cleavable oligomannosidic *N*-glycans in the Golgi apparatus [PDL1_His_(DG)] (Izadi et al. 2023b). CBB staining of purified proteins clearly shows the glycosylation-dependent size shift of PDL1_His_(DG) and PDL1_His_(Sia) when compared to PDL1_His_ (Fig. 2B). The glycosylation status of plant-made PDL1_His_ glyco-variants was first assessed by immunoblotting with a carbohydrate-recognizing lectin. In SDS-PAGE analysis, only PDL1_His_ (Sia) shows reactivity to the *N*-glycan-specific α2,6NeuAc-binding *Sambucus nigra* agglutinin (SNA) (Fig. 2C). Next, LC–ESI–MS glyco-profiling revealed that PDL1_His_ expressed in *N. benthamiana* ∆XF is mainly decorated with complex neutral glycans (GnGn); mannosidic and hybrid glycans (Man4Gn, Man5Gn) are only found on N192 and N219 is mostly unglycosylated, as previously reported (Izadi et al. 2024) (Fig. 2D and Fig. S2). In PDL1_His_ (Sia), the majority of complex glycans were efficiently elongated with terminal α2,6-sialic acid (NaNa). Hybrid glycans at N192 were also sialylated (Man4Na, Man5Na) and N219 remains unglycosylated (Fig. 2D and Fig. S2). *In planta* deglycosylation of PDL1_His_ was efficient in N35 and N200, but not complete in N192, where up to 40% of high-mannose glycans are detected (Fig. 2D and Fig. S2). High mannosidic glycans are also detected on N192 from several human melanomas (Morales-Betanzos et al. 2017). The fact that this site is decorated with hybrid glyco-structures indicates that it is probably not fully accessible to Golgi resident glyco-processing enzymes. It seems that the addition of kifunensine efficiently induced the accumulation of mannosidic *N*-glycans, but these were probably not accessible to be cleaved by EndoH in the Golgi apparatus. These results also explain the double band observed for PDL1_His_(DG) which probably correspond to fractions of deglycosylated protein and fractions with high-mannose glycans (Fig. 2B).

### *In planta* PD1-Fc/PDL1 interaction

**Fig. 3:**
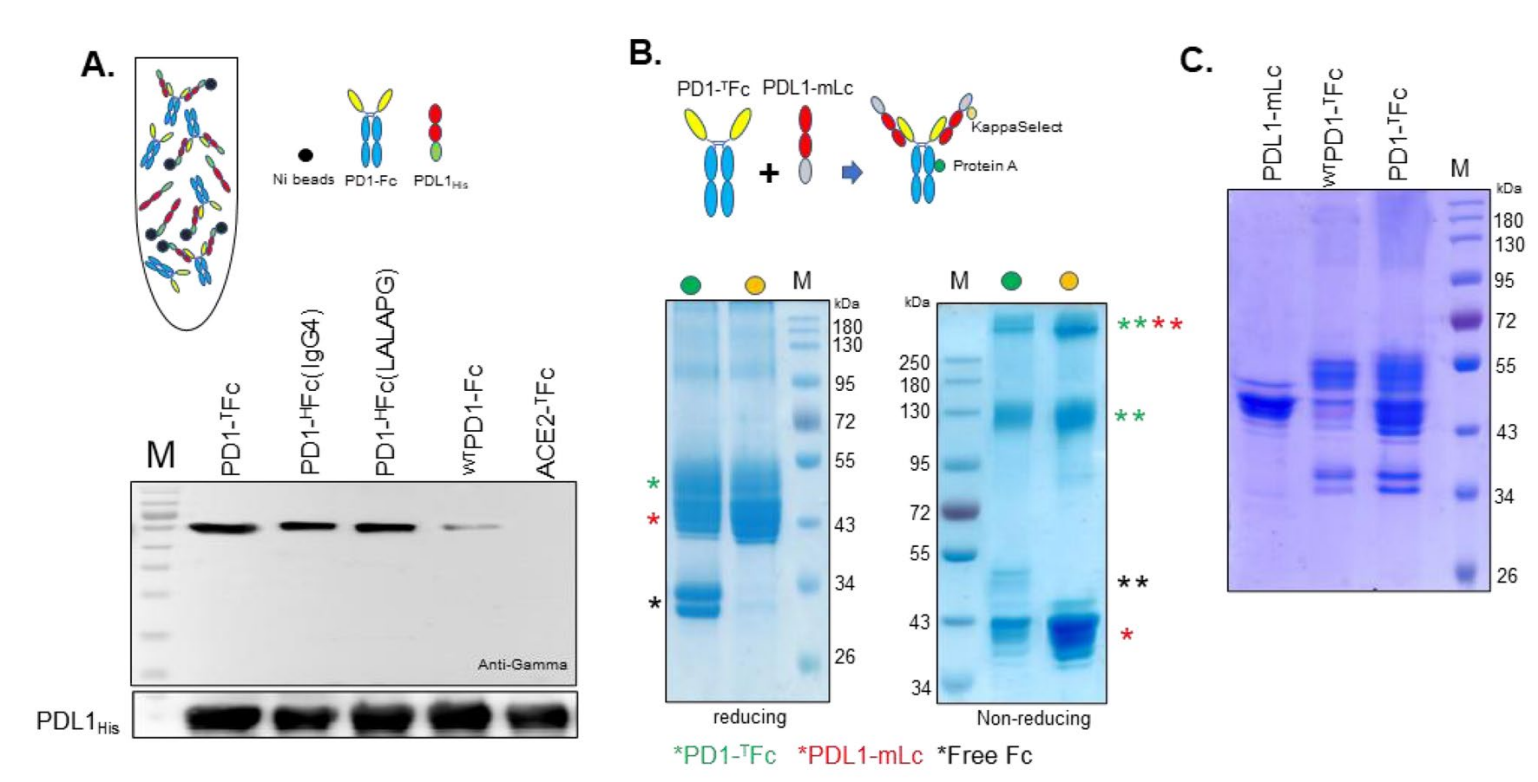
Interaction of PDL1 with PD1-Fc fusions. **A** Schematic representation of the immunoprecipitation assay (top). Western blot analysis under reduced conditions of co-purified proteins with anti-gamma and anti-His antibodies detecting PD1-Fc and PDL1_His_, respectively. An unrelated Fc fusion was used as negative control. **B** Schematic representation of the co-expression and purification strategy. Protein A with affinity to the Fc and KappaSelect with affinity to the Lc allow the simultaneous purification of PD1-Fc and PD1-mLc when they are interacting *in planta* (top). CBB staining of SDS-PAGE under reducing (bottom left) and non-reducing (bottom right) conditions of proteins purified with protein A (green dot) or KappaSelect (orange dot). **C** CBB staining of reducing SDS-PAGE of PDL1-mLc purified with kappaSelect (lane1) and of PDL1-mLc co-expressed with either ^WT^PD1-^T^Fc (lane 2) or PD1-^T^Fc (lane 3) purified with protein A. The apparent molecular mass of marker proteins (M) is shown in kilo Dalton (kDa). For more detailed information see Fig. S3

The binding of PD-Fc variants to PDL1_His_ was assessed in co-immunoprecipitation assays. Apoplastic fluid collected from leaves infiltrated with PDL1_His_ was spiked with ~ 3 µg of dimeric ^WT^PD1-Fc and PD1-Fc variants. After purification with Ni–NTA His•Bind beads, captured proteins were analyzed by immunoblotting with anti-His and anti-gamma antibodies. Figure 3A shows that all PD1-Fc formulations were efficiently co-purified with PDL1_His,_ but the binding affinity of ^WT^PD1-Fc was significantly lower.

Next, we evaluated the interaction of PD1-^T^Fc and PDL1-mLc *in planta*. The process relies on synchronous co-infection and co-replication of two viral vectors, each expressing a separate protein. PD1-^T^Fc and PDL1-mLc, derived from two different plant viruses (TMV and PVX), were found to be noncompeting and, thus, allowing for the efficient expression of hetero-oligomeric proteins (Fig. 3B). After co-expression of PD1-^T^Fc and PDL1-mLc, protein A-purified proteins (affinity to Fc domain) or kappaSelect (affinity to mLc) were analyzed by SDS-PAGE and CBB staining. Reduced SDS-PAGE of protein A purifications shows the presence of a ~ 45-kDa protein band in addition to the full-length PD1-^T^Fc fusion (~ 52-kDa) and free Fc (~ 34-kDa), while for kappaSelect only the ~ 45-kDa band and full-length PD1-Fc fusion (~ 52-kDa) are detected (Fig. 3B). In non-reducing conditions, we observed three protein bands at 45-, 130-, and > 250-kDa with an additional 50-kDa band detected only with protein A purification (Fig. 3B). Immunoblotting analysis revealed that the 50-kDa band detected only with anti-gamma antibodies represents dimers of free Fc purified via protein A (Fig. S3A). The 45-kDa (detected with anti-kappa antibodies) and the 130-kDa (detected with anti-gamma antibodies) bands are present in both purifications and correspond to disassociated monomeric PDL1-mLc and dimeric PD1-^T^Fc, respectively. Finally, the > 250-kDa protein band reacting in both purifications to anti-kappa and anti-gamma antibodies denotes oligomeric proteins resulting from the interaction between PD1-^T^Fc and PDL1-mLc (Fig. S3A). Using the same experimental settings (protein A purification), we compared the *in planta* interaction of PD1-^T^Fc and ^WT^PD1-^T^Fc to PDL1-mLc. The results show that the intensity of the 45-kDa band corresponding to co-purified PDL1-mLc is significantly lower when co-expression is performed with ^WT^PD1-^T^Fc (Fig. 3C and Fig. S3B).

### Binding of PD1-Fc variants to recombinant PDL1

The binding affinity of the different PD1-Fc variants to PDL1_His_(ΔXF) (GnGn glycosylation variant) was compared using ELISA. The results show that the binding affinity of PD1-^T^Fc is significantly stronger (EC50: 0.07 nM) than PD1-^H^Fc(LALAPG) and PD1-^H^Fc(IgG4) that show similar affinity to PDL1_His_ (EC50: ~ 0.3 nM). All three dimeric PD1-Fc fusions have a higher EC50 compared to the monomeric fusion PD1-^L^Fc(IgG4) (EC50: 2.2 nM), which can be explained by the increased avidity. Interestingly, removal of *N*-glycans in PD1-^T^Fc(DG) seems to significantly impair the binding to PDL1_His_ (EC50: 0.65 nM), which shows a ~ tenfold reduced affinity compared to its glycosylated counterpart, PD1-^T^Fc (Table 1A and Fig. S4A). The binding affinity of ^WT^PD1-^T^Fc and ^WT^PD1-Fc(HEK) using the same experimental settings was negligible. Despite the use of optimized settings (plates coated with 1 µg PDL1_His_ and serial dilutions starting at 20 µg), the binding of ^WT^PD1-^T^Fc and ^WT^PD1-Fc(HEK) to PDL1 remained up to 260-fold lower than PD1-^T^Fc (Table 1B and Fig. S4B). These results support the hypothesis that an optimized high-affinity analog of ^WT^PD1 can be used as a much more potent blockade/inhibitory molecule compared to the lower affinity of the native protein.

**Table 1:** Half maximal effective concentration (EC_50_) values in ELISA binding assays

(A) Binding of plant-produced PD1-Fc fusion variants to recombinant PDL1_His_			
Formulation	EC50 (ng/mL)	MW (mg/µmol)	EC50 (nM)
PD1-^T^Fc	6.2 ± 0.5	83	0.07
PD1-^H^Fc(IgG4)	23.1 ± 8	84	0.28
PD1-^H^Fc(LALAPG)	29.8 ± 13	83	0.36
PD1-^T^Fc(DG)	54.1 ± 19	83	0.65
PD1-^L^Fc(IgG4)	87.6 ± 0.17	40	2.20

(B) Binding of ^WT^PD1-^T^Fc and ^WT^PD1-Fc(HEK) to recombinant PDL1_His_. An unrelated Fc fusion protein was used as negative control		
Formulation	EC50 (ng/mL)	EC50 (nM)
^WT^PD1-^T^Fc	1876	22.6
^WT^PD1-^H^Fc(HEK)	453.3	5.33

(C) Binding of PD1-^T^Fc to PDL1_His_ glyco-variants. An unrelated His-tagged protein was used as negative control.			
PD1-^T^Fc	PDL1_His_ (GnGn)	PDL1_His_ (Sia)	PDL1_His_ (DG)
EC50 (ng/mL)	9.1 ± 2.6	4.8 ± 2.5	10.5 ± 1.2
EC50 (nM)	0.11	0.06	0.13

PDL1His(GnGn): PDL1_His_ expressed in ΔXF plants; PDL1_His_(Sia): PDL1_His_ co-expressed in ΔXF plants together with the necessary genes for protein sialylation; PDL1_His_(DG): PDL1_His_ co-expressed in ΔXF plants with EndoH in the presence of kifunensine. Data represent the mean ± SD values of triplicates

It has been shown that the extensive glycosylation of PDL1 hinders its detection by anti-PDL1 antibodies and can lead to inaccurate readout from a variety of bioassays (Lee et al. 2019)**.** Here, we assess if PDL1_His_ glycosylation affects the binding of the high-affinity PD1-^T^Fc variant. Plates coated with plant-made PD1-^T^Fc were probed with a serial dilution of PDL1_His_ glyco-variants. The similar EC50 values for the PDL1_His_(GnGn), PDL1_His_(Sia) and PDL1_His_(DG) (Table 1C and Fig. S4C) show that plant-made PD1-^T^Fc lacks any preference towards unglycosylated PDL1. Importantly, the binding of PD1-^T^Fc to PDL1 seems not to be affected by protein sialylation, a common alteration of cancer receptors that play a key role in the acquisition of molecular resistance to diagnostic and targeted therapeutic agents (Zhou et al. 2021; Duarte et al. 2021; Rodrigues et al. 2021a, 2021b).

Finally, we used surface plasmon resonance (SPR) to compare the binding kinetics of PD1-^T^Fc, ^WT^PD1-Fc(HEK) and avelumab (Bavencio^®^), a PDL1 therapeutic IgG1 antibody. The results show that PD1-^T^Fc and avelumab bind with nanomolar affinity to PDL1 (K_D_ 2.3 nM and 1 nM, respectively) with comparable association and dissociation rates (*k*_*a and*_* k*_*d*_). To analyze the binding kinetics of ^WT^PD1-Fc(HEK), we optimized the conditions using a higher concentration of the serial dilutions (250 nM-4000 nM). Even with this setting, it was not possible to fit the curve kinetically and determine the *k*_*a and*_* k*_*d*_. Nevertheless, the K_D_ value from the steady-state analysis was in the micromolar range (K_D_ 2.4 µM) (Table 2 and Fig. S5).

**Table 2:** Binding kinetics of PD1–^T^Fc, ^WT^PD1–Fc(HEK) and avelumab to PDL1_His_

Formulation	ka1 (1/Ms)	kd1 (1/s)	kD(M)	Rmax (RU)
PD1-^T^Fc	10E5 ± 3E4	2.3E-4 ± 2E-5	2.3E-9 ± 4E-11	113 ± 1
^WT^PD1-^H^Fc(HEK)	np	np	2.4E-6 ± 4E-8	90 ± 0.7
Avelumab	4E5 ± 3E3	4E-4 ± 6E-6	9.4E-10 ± 2E-11	79 ± 0.4

Kinetic parameters such as association and dissociation rates (*k*_*a and*_* k*_*d*_), dissociation equilibrium constants (K_D_), and binding response (expressed as response units or RUs) were used to assess the affinity of plant-made PD1-^T^Fc, HEK-derived ^WT^PD1-Fc and therapeutic avelumab (Bavencio^®^) to recombinant PDL1_His_(ΔXF). Values are given as mean of three runs (see figure S5). Data represent the mean ± SD values of triplicates

*np* not possible

### Binding of PD1-Fc variants to cell surface PDL1

**Fig. 4:**
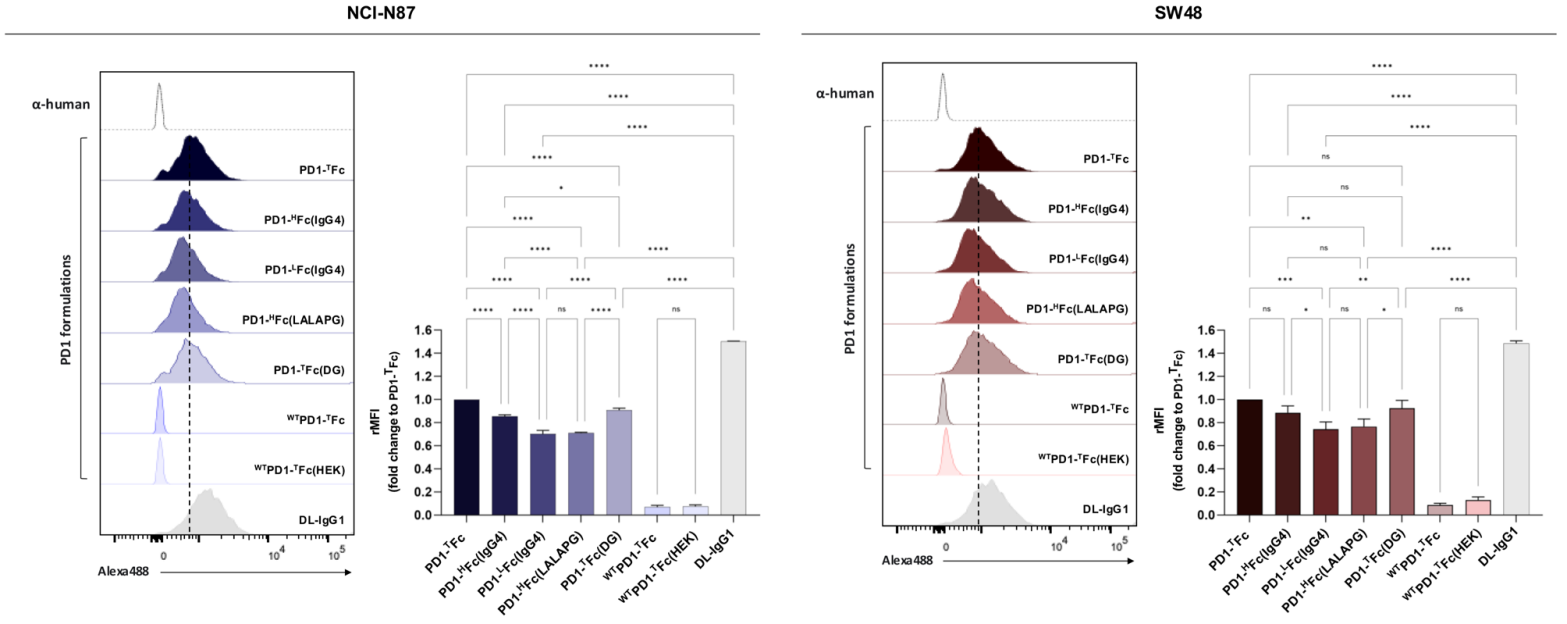
Binding of PD1-Fc variants to PDL1-expressing gastrointestinal cancer cells. In vitro IFNγ stimulation induces the cell surface expression of PDL1, allowing the assessment of the binding of PD1-Fc variants in NCI-N87 and SW48 gastrointestinal cancer cell lines, via flow cytometry. Plant-derived durvalumab and secondary α-human antibody were used as positive and negative controls, respectively. Depicted histograms are representative of three independent experiments. ****p < 0.0001; ***p < 0.001; **p < 0.01; *p < 0.05; ns: not significant. PD1-^T^Fc mutated HAC-V PD1 fused to a truncated hinge region, DL-IgG1 durvalumab, ^WT^PD1-^T^Fc wild-type PD1 receptor fused to a truncated hinge region, PD1-^H^Fc(LALAPG) and PD1-^H^Fc(IgG4) PD1 fused to mutated Fcs lacking Fcγ receptor engagement, PD1-^L^Fc(IgG4) PD1 fused to the Fc-IgG4 via a linker of glycine and serine, PD1-^T^Fc(DG) PD1 carrying a single GlcNAc residue following *in planta* deglycosylation, ^WT^PD1-Fc(HEK) PD1-Fc fusion expressed in HEK293 cells

It has been shown that the steric hindrance of PDL1 glycosylation significantly affects antibody recognition, with implications in patient therapeutic stratification (Lee et al. 2019). Therefore, we evaluated the ability of PD1-Fc formulations to bind to cell surface PDL1 expressed by two gastrointestinal cancer cells. The metastatic gastric carcinoma cell line NCI-N87 and the colorectal carcinoma cell line SW48 have low basal expression of PDL1, as demonstrated by flow cytometry (Fig. 4). Following in vitro interferon gamma (IFNγ) stimulation, both cell lines start expressing similar PDL1 levels at the cell surface in more than 80% of cells.

Fluorescence-activated cell sorting (FACS) shows that, compared to PD1-^T^Fc, the binding of both plant- and HEK-derived ^WT^PD1-^T^Fc is negligible. For both cells lines, the binding of PD1-^H^Fc(LALAPG) is significantly lower than PD1-^T^Fc and comparable to the monomeric fusion PD1-^L^Fc(IgG4), while differences in binding of PD1-^H^Fc(IgG4) and deglycosylated PD1-^T^Fc(DG) are only significant in NCI-N87 cells. Importantly, in both cell lines, the binding of plant-made durvalumab (DL-IgG1, Izadi et al. 2024) is significantly higher than PD1-^T^Fc (~ 1.5-fold) (Fig. 4).

### Blocking of PD1/PDL1 interaction by PD1-Fc variants

**Fig. 5:**
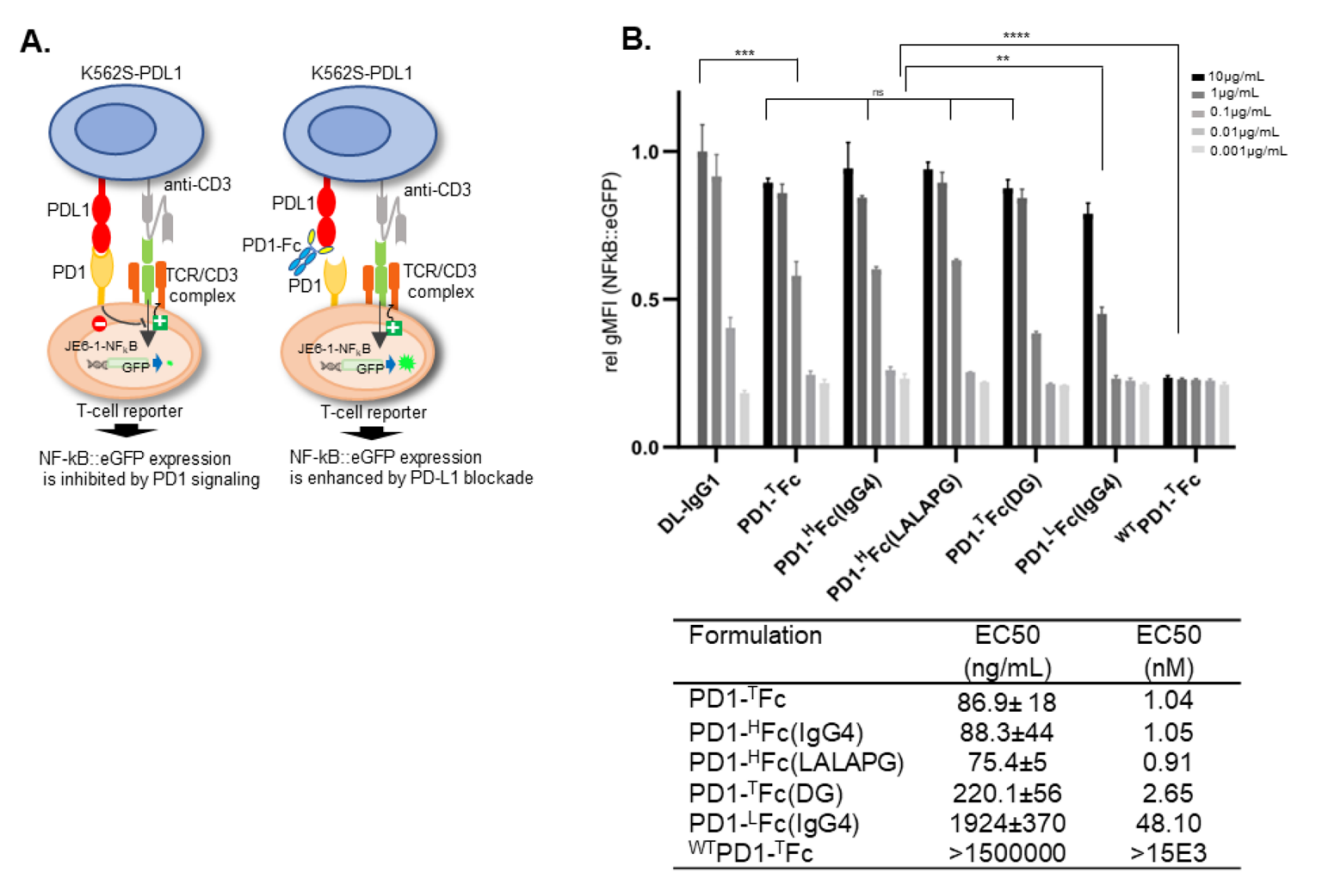
Blocking of PD1/PDL1 interaction by PD1-Fc variants. **A** Simplified schematic representation of the use of a transcriptional PD1^+^NF-κB::eGFP reporter T-cell line to evaluate the impact of PD1-Fc variants on T-cell activation (adapted from Sharma et al. 2024). **B** PD1 expressing NF-κB::eGFP reporter cells were stimulated for 24 h with K562S-PDL1 in presence of PD1-Fc variants. The PDL1 antibody avelumab (3 µg/mL) was assumed to mediate full blockade of PDL1 and the gMFI (NF-kB::eGFP value) obtained in the presence of this antibody was set to 1. PD1-Fc variants were used in tenfold dilution steps starting at 10 µg/mL. For comparison, plant-derived durvalumab (DL-IgG1) was also included in experiments. Data are derived from two independent experiments performed in triplicate (*n* = 6). Inhibition curves (Fig. S6) and half maximum effective concentrations (EC_50_) were calculated from normalized data using a four-parameter logistic function. ****p < 0.0001; **p < 0.01; *ns* not significant

Next, we assessed the inhibitory properties of PD1-Fc variants against PD1/PDL1 complex at the cellular level. We have previously used a PD1 reporter T-cell line (JE6-1-NF-kB::eGFP-PD1) to evaluate the antagonistic capacity of plant-made durvalumab variants in a functional assay (De Sousa Linhares et al., 2019; Izadi et al. 2024). Cross linking of the TCR-CD3 complex with CD3-antibody fragments expressed on K562 based stimulator cells (K562S-PDL1) results in a strong expression of the eGFP reporter gene. Negative co-stimulatory signals induced by engagement of PD1 with K562S expressing PDL1 lead to the inhibition of TCR/CD3 signaling and inhibition of eGFP-expression. PD1-Fc targeting PDL1 should block PD1 engagement and restore eGFP expression (Fig. 5A).

As with PDL1 antibodies (De Sousa-Linhares et al., 2019), dose–response curves and half maximal effective concentrations (EC_50_) provided valuable information to predict the functional potency of PD1-Fc fusions as decoy/trap molecules able block the PD1/PDL1 interaction. Plant-produced PD1-Fc formulations efficiently reverted PD1 mediated reporter inhibition in a dose-dependent manner (Fig. 5B and Fig. S6). All dimeric PD1-Fc show similar blocking potency (EC_50_ up to 1 nM) including the deglycosylated PD1-^T^Fc(DG)(EC_50_ 2.65 nM), which is significantly higher than the blocking obtained with the monomeric fusion (PD1-^L^Fc(IgG4, EC_50_ 48.1 nM). The blocking capacity of ^WT^PD1-^T^Fc with EC50 in the micromolar range (~ 15.000-fold lower) agrees with its lower binding affinity to PDL1.

## Discussion

Despite PD1/PDL1 blockade representing a significant advancement in cancer mono- or combination immunotherapeutic setting, molecular resistance remains a major obstacle as only 20–40% of patients benefit from these therapies. Many tumors do not respond well to this treatment, and merely blocking the immune suppression pathways by checkpoint antibodies may not render optimal tumor growth inhibition. Several potential resistance mechanisms have been proposed, including abnormal antigen expression and presentation (Nowicki et al. 2018). Aberrant glycan structures play key roles in the acquisition of molecular resistance to targeted therapeutic agents and there is emerging evidence linking PDL1 glycosylation to cancer immunotherapy efficacy (Wang et al. 2020). Since PDL1 glycosylation is required for its physical interaction with PD1, antibodies targeting PDL1 glycans may improve the therapeutic efficacy in the clinic (Li et al. 2018a; Benicky et al. 2021).

Binding of the hinge-Fc region to FcγRs has been shown to exert a profound impact on antibody function and in vivo efficacy (Chen et al. 2019b). Most antibodies targeting the PD1/PDL1 interaction lack the ability to trigger ADCC either because they possess a modified Fc region (durvalumab and atezolizumab) or belong to the IgG4 subclass (pembrolizumab, nivolumab, and cemiplimab), to obviate Fc-mediated cytotoxic effects on T cells. While PD1 is mainly expressed on T cells, PDL1 is frequently overexpressed by tumors or the tumor-associated microenvironment and, therefore, a clinical validated cancer biomarker. Although the optimal IgG scaffold for antibodies targeting PDL1 is still being debated, it is possible that the current PDL1 therapeutic mAbs may not optimally harness FcγR pathways and enhanced ADCC could provide additional therapeutic potential by promoting potent and selective tumor killing (Chen et al. 2020; Jin et al. 2021; Cohen Saban et al. 2023). Therefore, thorough investigation of ICIs regarding their effector functions and impact on therapeutic efficacy has remained imperative in recent years.

In addition to the transmembrane forms, soluble PD1 and PDL1 can be generated by protease cleavage and accumulate in blood (Zhu and Lang 2017). Although the function of these circulating proteoforms remains unclear, it has been reported that soluble PDL1 retains inhibitory activity and is associated with advanced disease and worse prognosis. A recent study showed that a secreted PDL1 splicing variant resisted PDL1 antibody treatment and that this decoying function of PDL1 variants may be one of the reasons for cancers being resistant to anti-PDL1 therapy (Sagawa et al. 2022). Using a similar rationale, soluble PD1 can block the natural PD1/PDL1 interaction (Shin et al. 2013; Liu et al. 2015).

The use of plants can significantly accelerate biologics production, with relatively lower infrastructure costs, compared with mammalian cell-based manufacturing. Novel technological approaches and genome engineering tools have streamlined plant-based expression processes and contributed to the generation of more efficient and productive plant strains with several biologics successfully being used in clinical trials and treatment of patients (Zahmanova et al. 2023).

Here, we investigate the potential of plant molecular farming to generate soluble engineered PD1s and assess their ability to bind to PDL1 expressed recombinantly, or endogenously in cancer cells. In addition, we determined if plant-made PD1 could be used as a bait/trap to block PDL1 from interacting with its natural receptor in cell-based assays. PD1 was expressed in plants as fusions to Fc domains from different IgG-based scaffolds. Recently, we used different IgG scaffolds to generate durvalumab formulations with different affinity to Fc receptors (Izadi et al. 2024). According to our previous data, PD1-^T^Fc should retain the ability to bind to FcγRs, while PD1-^H^Fc(LALAPG) and PD1-^H^Fc(IgG4) should have reduced affinity for these receptors. This could be advantageous, since targeted blockade or genetic depletion of the inhibitory FcγRIIB receptor have been used to overcome therapeutic resistance and boost activity of antibodies in cancer immunotherapy (Teige et al. 2019; Cohen Saban et al. 2023). In addition, the M252Y/S254T/T256E (YTE) triple mutation should increase the binding of PD1-^H^Fc(LALAPG) to the neonatal receptor (FcRn) (Dall’Acqua et al. 2002) and the E233P/F234V/P235A (PVA) amino acid substitutions in PD1-^H^Fc(IgG4) reduces its ability to bind to FcγRI (Zhang et al. 2018).

All PD1-Fc fusions are efficiently expressed in plants. However, instabilities created by dimerization lead to the proteolytic cleavage of the Fc domain. Although protein degradation can be solved by the generation of monomeric Fc fusions (Gattinger et al. 2021), these have reduced avidity and lack FcγR affinity. Fusion of PD1 to Fc seems to contribute to overall protein stability, since all the attempts to express a monomeric histidine tagged PD1 and ^WT^PD1 were unsuccessful. Nevertheless, Fc domain cleavage is a recurrent shortcoming of plant-made Fc fusions that needs to be addressed in future investigations.

PD1 glycosylation is critical for maintaining its stability, but its significance on the interaction to PDL1 is not fully elucidated. Although structural analysis of PD1 revealed that all PD1 glycosylation sites are located away from the binding site of PD1/PDL1 (Chen et al. 2019a), glycosylation of PD1, especially at the N58 site, is essential for mediating its interaction with PDL1 (Sun et al. 2020). Here, we aimed to modulate the glycosylation profile of plant-made PD1-Fc in order to identify glycans that could potentially increase the binding of these decoy proteins to PDL1. However, the glyco-profiling of plant-made PD1-Fcs revealed unexpected results. When expressed in ∆XF plants, the Fc glycosylation site is decorated with complex glycans lacking plant specific glyco-epitopes as expected for secreted proteins, but the majority of the glycans decorating PD1 are of the high-mannosidic type. These immature unprocessed glycans contrast with those observed for ^WT^PD1-Fc(HEK), and are most likely due to a different structural conformation of the plant-made PD1-Fcs. These differential conformations are specific for the PD1 domain (wild-type and mutated), since we have previously reported on plant-made Fc fusions with fully processed complex glycans (Castilho et al. 2013, 2021; Izadi et al. 2021).

Importantly, plant-based transient expression of recombinant proteins can be used as a tool to quickly assess protein–protein interactions in vivo. Indeed, the co-expression of PDL1 with PD1-^T^Fc and ^WT^PD1-^T^Fc showed that they are able to interact *in planta* and allowed us to identify improved binders. Notably, from the three dimeric PD1-Fc fusions, PD1-^T^Fc (with Fc-mediated effector functions) showed the highest affinity to PDL1, both soluble or expressed at the cell surface of NCI-N87 gastric cancer cells, and PD1-^T^Fc deglycosylation only slightly decreases its binding. Interestingly, a similar observation was reported for plant-made durvalumab antibodies produced with same Fc formulations (IgG1, IgG1(LALAPG) and IgG4) (Izadi et al. 2024). Importantly, the ability to block the PD1/PDL1 interaction is similar to all dimeric PD1-Fc fusions.

Studies in triple-negative breast cancer (TNBC) overexpressing PDL1 showed that glycosylation potentiates its interaction with PD1 and promotes immune escape (Li et al. 2018a). Alterations in the glycosylation pattern of PDL1 expressed in cancer cells might be related to the acquisition of molecular resistance to targeted therapeutic agents (Benicky et al. 2021).

Clinically, PDL1 expression, quantified using immunohistochemistry assays, is used as a reference index of mAb treatment efficacy (Kumagai et al. 2020). Structural hindrance by *N*-glycan on PDL1 in fixed samples impedes its recognition by PDL1 diagnostic antibodies but the removal of *N*-linked glycosylation enhances PDL1 detection in a variety of bioassays (Lee et al. 2019; Dressler et al. 2022; Wang et al. 2020).

Here, we took advantage of the high tolerance of *N. benthamiana* for glycoengineering, to generate PDL1 decorated without and with tailored *N*-glycans including sialylation. The binding affinity of plant-made PD1-^T^Fc seems not be affected by the PDL1 glycosylation status. Plant-made affinity improved PD1-Fc proteins could therefore have utility for developing a robust assay system to test for PDL1 expression in tissue (since such fusion proteins would not be affected by aberrant PDL1 glycosylation).

Most importantly, we could demonstrate that plant-made affinity-enhanced PD1 fusion proteins are highly efficient in blocking PD1 engagement in protein-based but also in cell-based assays. Consequently, plant-expressed PD1-based ICIs can be considered as cost-effective alternative to antibodies targeting the PD1/PDL1 axis. PDL1 targeting PD1-Fc proteins with ADCC activity might have enhanced efficacy to combat tumors expressing high levels of PDL1. *N. benthamiana* plants constitute a highly efficient and versatile glycoengineering platform that can be exploited to develop PD1 fusion proteins with optimized cytotoxic effector function and reduced susceptibility to resistance mechanisms.

## Supplementary Information

Below is the link to the electronic supplementary material.

Supplementary file1 (PDF 1241 KB)

## Data Availability

The data supporting this article are included in the supplementary material.
