# Supplementary material for: An engineered PD1-Fc fusion produced in N. benthamiana plants efficiently blocks PD1/PDL1 interaction
Source: Plant Cell Rep. 2025 Mar 22;44(4):80. doi: 10.1007/s00299-025-03475-0 (PMC11929711; doi:10.1007/s00299-025-03475-0)
Supplement: Supplementary file 1 — Supplementary file1 (PDF 1241 KB) [file 299_2025_3475_MOESM1_ESM.pdf]

Supplementary figures

Fig. S1

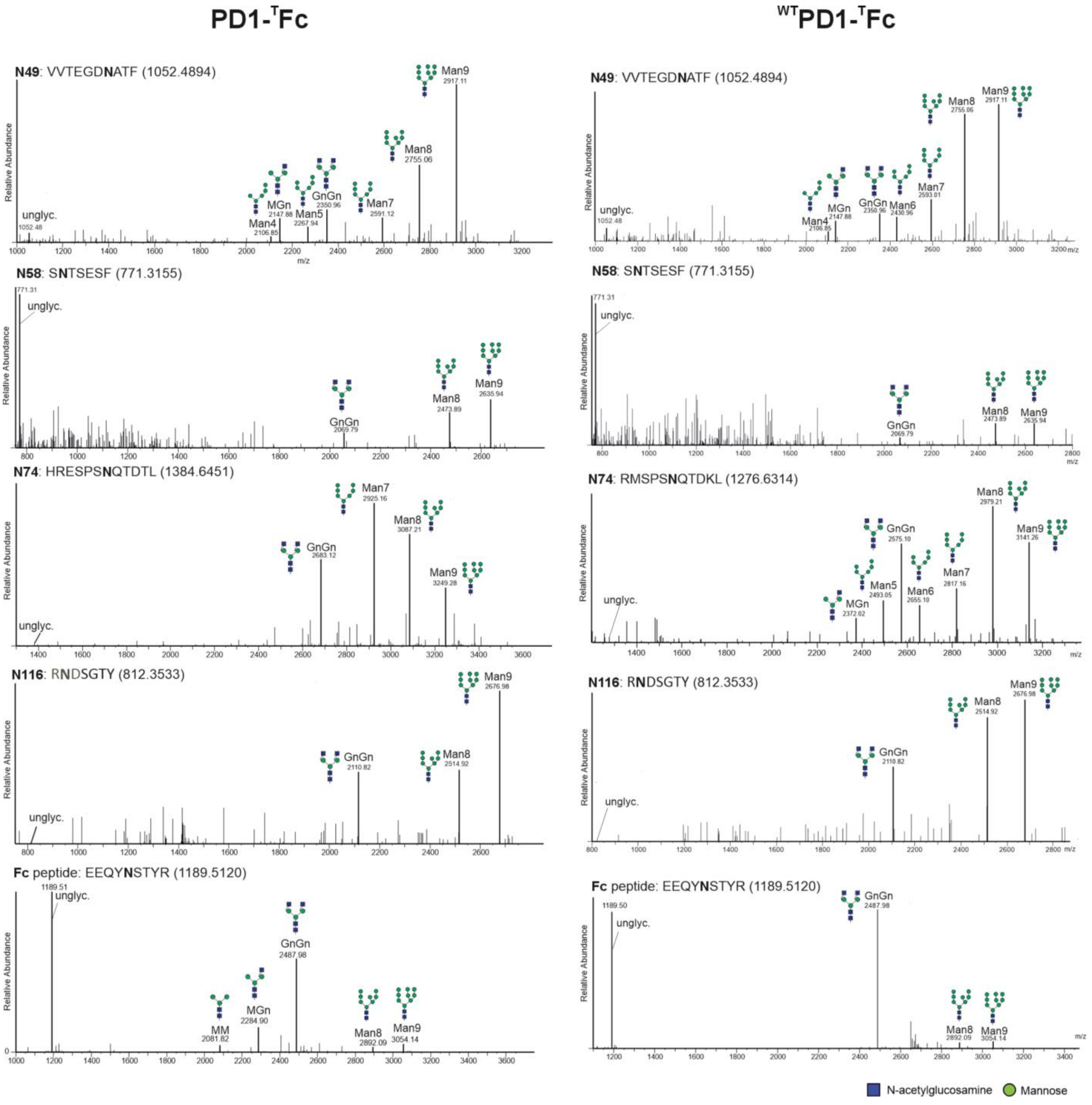

Figure S1 continue

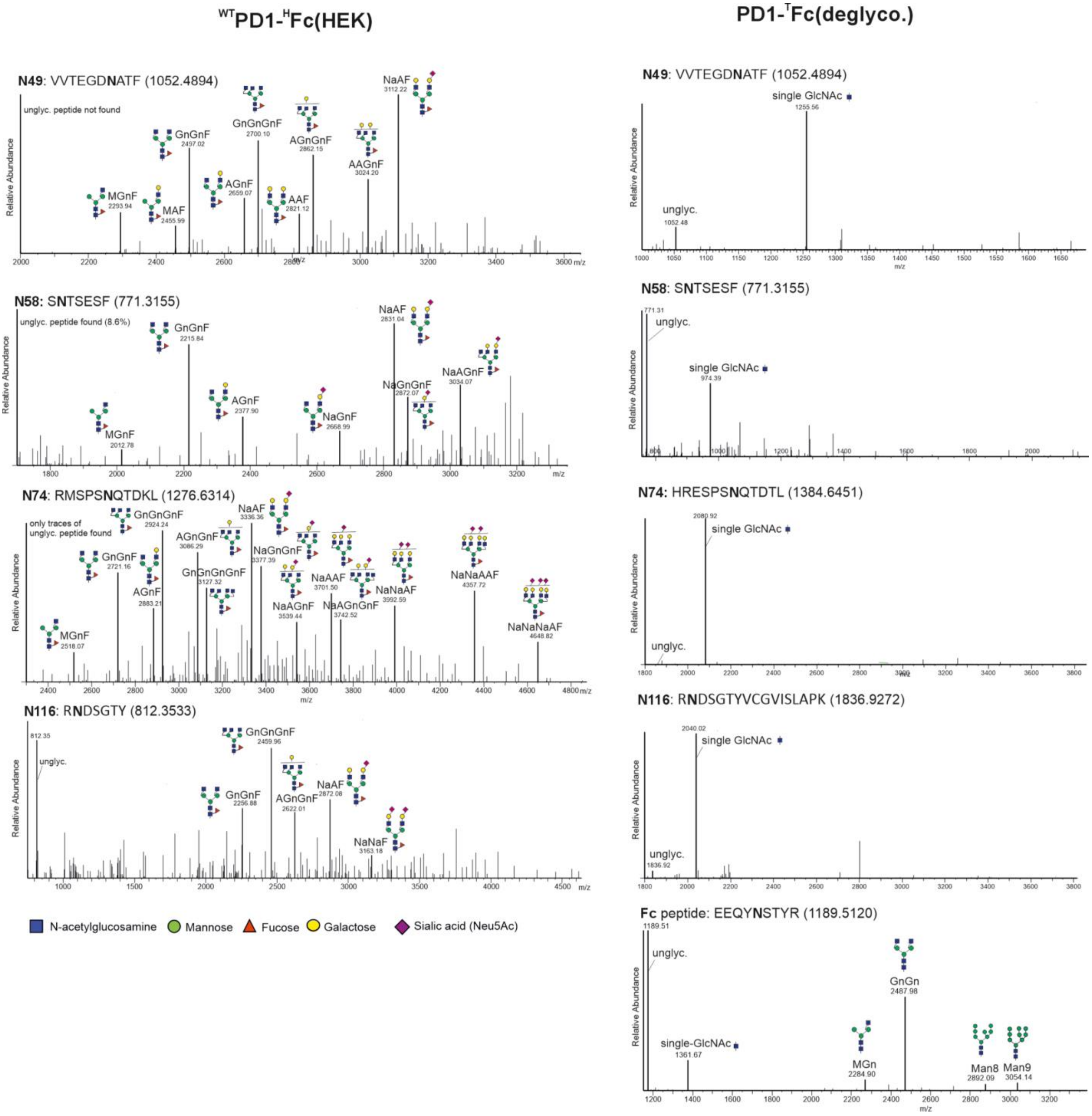

Fig. S1: Glycosylation profiles of WTPD1-Fc(HEK) and plant-derived PD1-Fc variants expressed in *N. benthamiana* ΔXF plants. The assigned N-glycan structures were labelled according to the ProGlycAn nomenclature. A cartoon illustration highlights the main glycan structures detected for each peptide. For details see http://www.functionalglycomics.org/.

Figure S2:

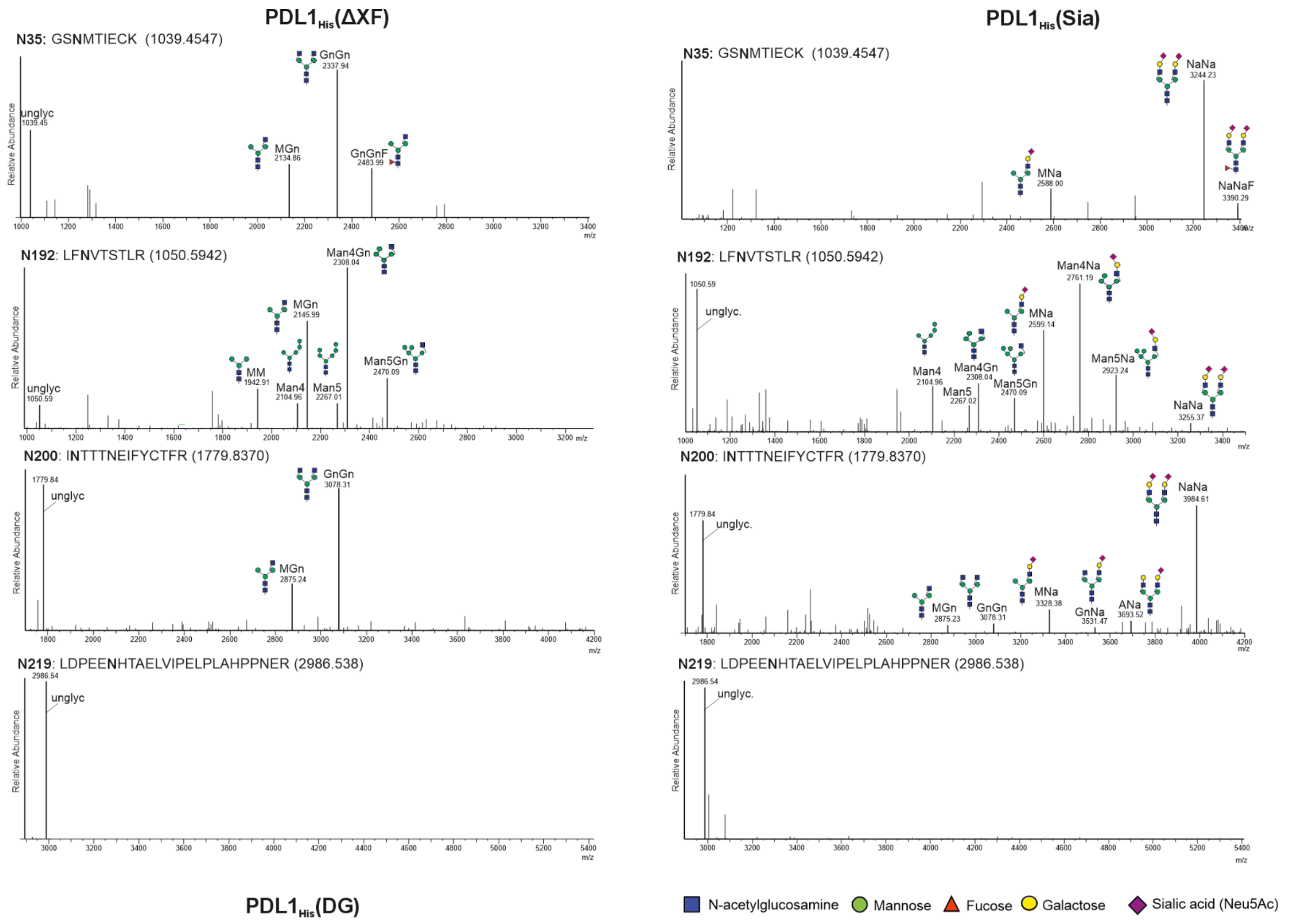

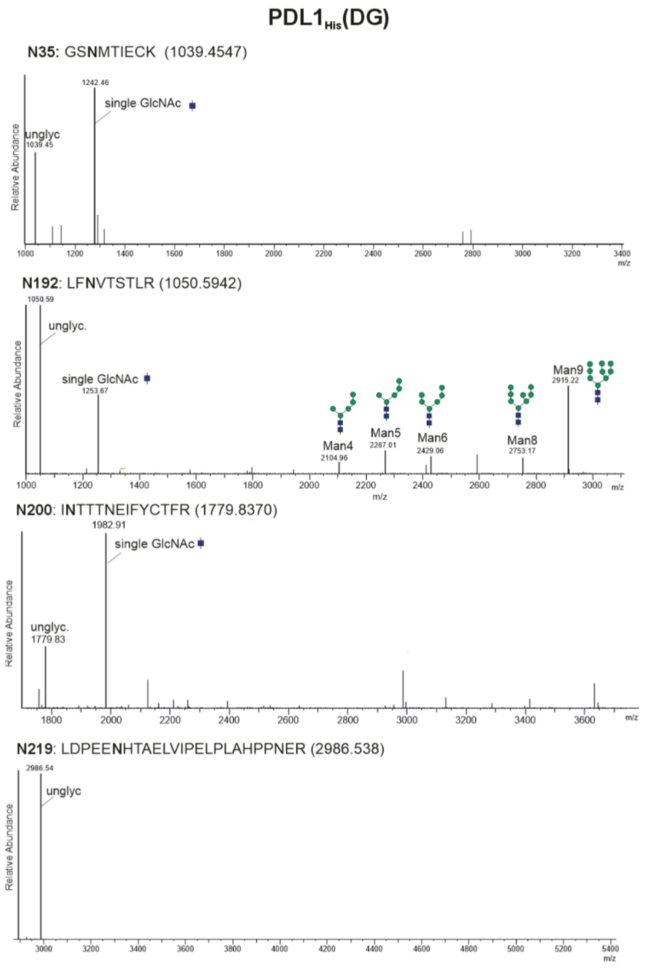

Fig. S2: Glycosylation profiles of plant-derived PDL1$_{His}$ glyco-variants. PDL1$_{His}$(ΔXF): PDL1$_{His}$ expressed in ΔXF plants. PDL1$_{His}$(Sia): PDL1$_{His}$ co-expressed in ΔXF plants together with the necessary genes for protein sialylation and PDL1$_{His}$(DG): PDL1$_{His}$ co-expressed in ΔXF plants with endoH in the presence of kifunensine. The assigned N-glycan structures were labelled according to the ProGlycAn nomenclature. A cartoon illustration highlights the main glycan structures detected for each peptide. For details see http://www.functionalglycomics.org/.

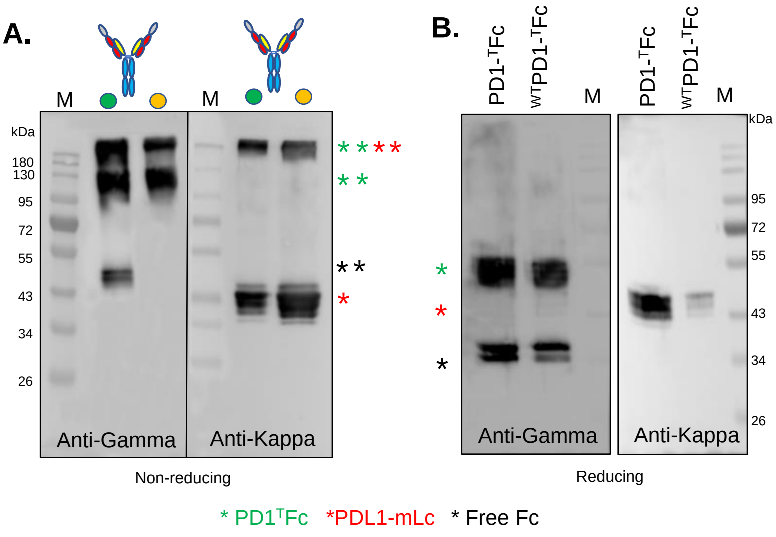

Fig. S3: In planta interaction of PDL1 and PD1-Fc fusions. (A) Western blot analysis of proteins purified with protein A (green dot) and KappaSelect (orange dot) after co-expression of PDL1-mLc and PD1-^{T}Fc. Co-purified proteins were analysed in non-reducing conditions with anti-gamma (left) and anti-kappa (right) antibodies. (**B**) Western blot analysis of proteins purified with protein A after co-expression of PDL1-mLc with PD1-^{T}Fc and ^{WT}PD1-^{T}Fc. Co-purified proteins were analysed under reducing conditions with anti-gamma (left) and anti-kappa (right) antibodies. The apparent molecular mass of marker proteins (M) is shown in kilo Dalton (kDa).

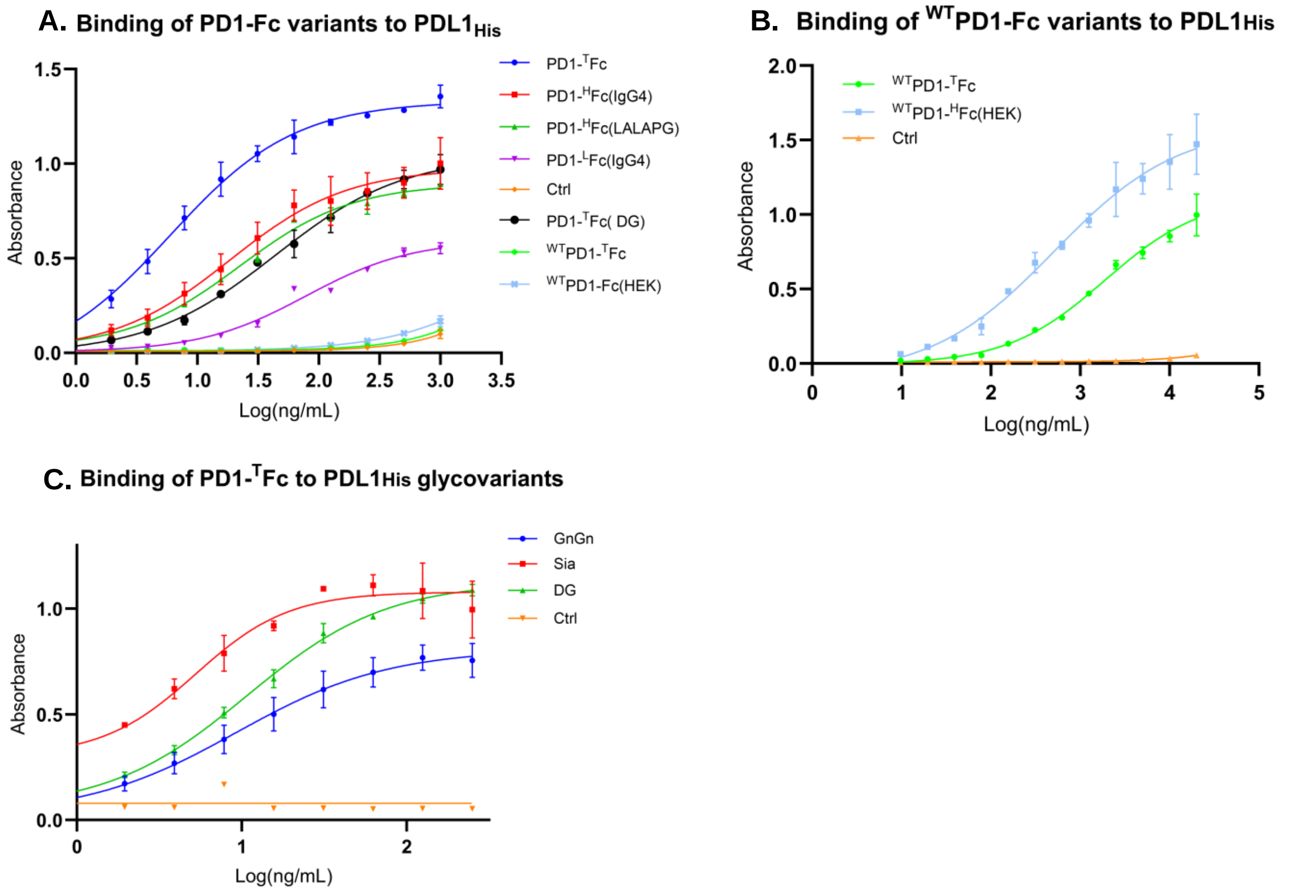

Fig. S4: ELISA binding assays. (**A**) Binding of plant-produced PD1-Fc fusion variants to recombinant $PDL1_{His}$. Serial dilutions of PD1-Fc variants (1000 to 0.48 ng/mL) were added to plates coated with 200 ng of $PDL1_{His}$ and detected with anti-human IgG-HRP. (**B**) Binding of ^{WT}PD1-^{T}Fc and ^{WT}PD1-Fc(HEK) to recombinant $PDL1_{His}$. Serial dilutions of PD1-Fc variants (20000 to 10 ng/mL) were added to plates coated with 1 µg of $PDL1_{His}$ and detected with anti-human IgG-HRP. An unrelated Fc fusion was used as negative control. Data represent the mean values of triplicates. (**C**) Binding of plant-produced PD1-^{T}Fc fusion to $PDL1_{His}$ glyco-variants: $PDL1_{His}$ expressed in ΔXF plants. $PDL1_{His}$(Sia): $PDL1_{His}$ co-expressed in ΔXF plants together with the necessary genes for protein sialylation and $PDL1_{His}$(DG): $PDL1_{His}$ co-expressed in ΔXF plants with endoH in the presence of kifunensine. An unrelated His-tagged protein was used as negative control. Data represent the mean values of triplicates. Bars represent SD.

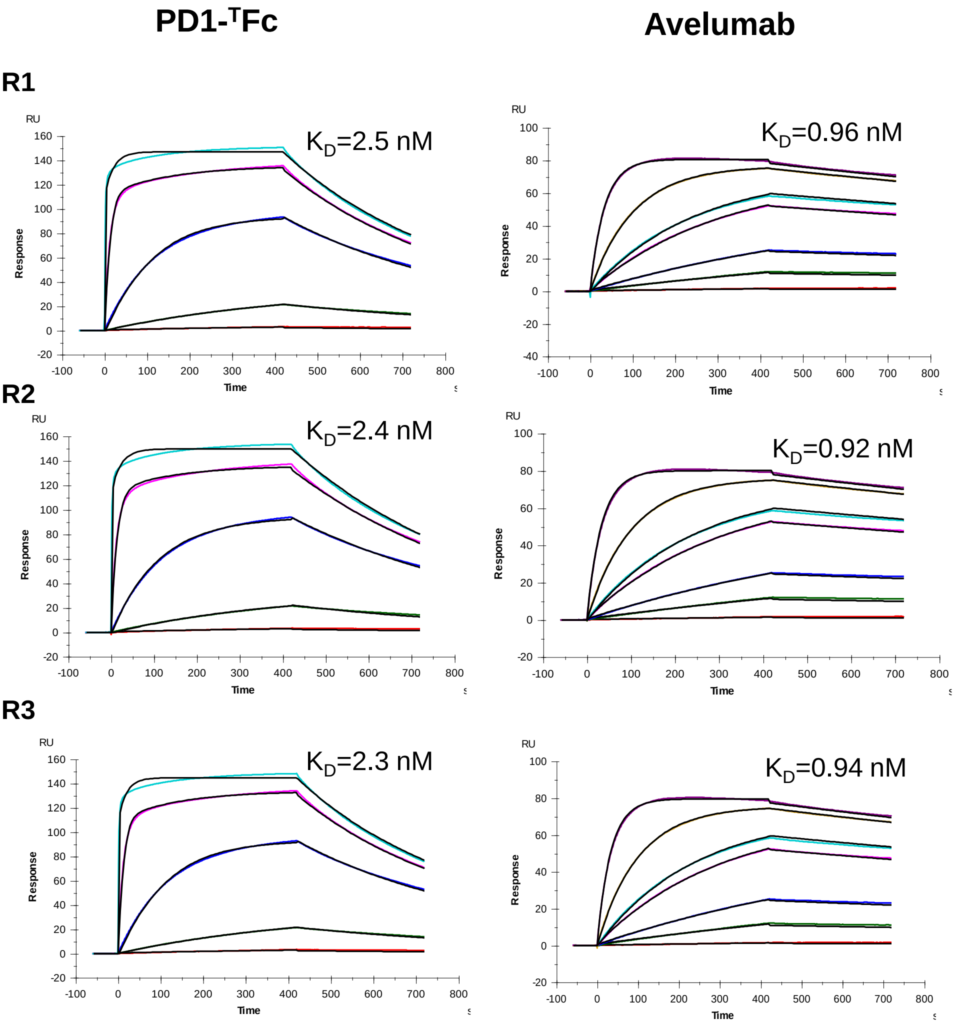

Fig. S5: SPR sensorgrams for the binding of PD1-^{T}Fc, ^{WT}PD1-Fc(HEK) and Avelumab to PDL1$_{His.}$ Analyte serial dilutions of 250 nM-4000 nM, 0.1 nM-1000 nM and 0.1 nM-75 nM were used to probe affinity of PDL1$_{His}$ to ^{WT}PD1-^{H}Fc(HEK), PD1-^{T}Fc and Avelumab, respectively. Experiments were done in three runs (R1-R3). K_D values are given for each sensorgram.

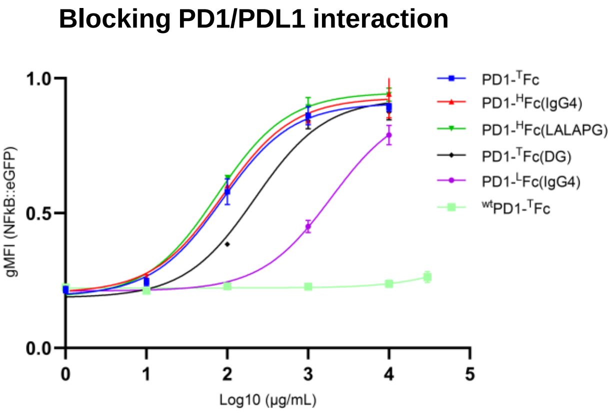

Fig. S6: Blocking PD1/PDL1 interaction by PD1-Fc variants. Inhibition curves used to determine functional half maximum effective concentrations (EC_{50}). PD1-Fc variants were evaluated at different concentrations (ranging from 10 to 0.001 µg/mL) using PD1^{+}NF-κB::eGFP T-cell reporters co-cultured with T-cell stimulator cells expressing PDL1. gMFI: geometric mean of fluorescence intensity. Data are derived from two independent experiments performed in triplicates (n=6). Bars represent SD.
